# Parasites of Some Freshwater Fish from Armand River, Chaharmahal va Bakhtyari Province, Iran

**Published:** 2012

**Authors:** M Raissy, M Ansari

**Affiliations:** 1 Department of Aquatic Animal Health, Faculty of Veterinary Medicine, Islamic Azad University–Shahrekord Branch, Iran; 2 Young Researchers Club, Islamic Azad University–Shahrekord Branch, Iran

**Keywords:** Fish Parasites, Species, Epidemiology, Iran

## Abstract

**Background:**

The aim of this study was to detect the occurrence of parasites in fish in Armand River, Chaharmahal va Bakhtyari Province regarding the importance of native fish population in the river.

**Methods:**

The occurrence of parasites was investigated in 6 native fish (*Capoeta capoeta, C. damascina, C. aculeta, Barbus barbulus, B. grypus* and *Glyptothorax silviae*) collected from the current main channel of the river from autumn 2009 to summer 2010.

**Results:**

63.7% of the studied fishes were infected with 19 parasite species including *Ichthyophthirius multifiliis, Myxobolus musayevi, Dactylogyrus lenkorani, D. gracilis, D. pulcher, D. chramuli, D. akaraicus, D. skrjabiensis*, two species of *Gyrodactylus, Paradiplozoon sp., Lamproglena compacta, Copepodid of Lernaea cyprinacea, Ergasilus* sp., *Allocreadium isoporum, Allocreadium pseudaspii, Kawia* sp., *Bothriocephalus gowkongensis and Rhabdochona denudata*. The infection rate was significantly higher (P<0.05) in *C.aculeata* while the maximum parasite diversity was found in C.damascina. The infection rate was also significantly different in four seasons (P<0.05) but no significant differences were found among fishes with different weight and length.

**Conclusion:**

High prevalence of *Ichthyophthirius multifiliis* and *Rhabdochona denudata* may affect native fish population. Monogenean parasites *Dactylogyrus akaraicus* and *D. skrjabiensis* collected from *B. barbulus* and *C. capoeta* are reported for the first time in Iran. *B. barbulus* is also reported as a new host for aforementioned parasites.

## Introduction

Parasites in fish have been a great concern since they often produce disease conditions in fish which will lead reduced growth, increase in the fishes’ susceptibility to other diseases as well as fish loss. The study on parasites of freshwater fish in Iran dates back to 1949, when Bychowsky reported three *Dactylogyrus* species and one *Ancyrocephalus* on the gills of fishes in Karkheh River (1). Since then many endo and ectoparasites has been reported from Iranian freshwater fish as the records reached over 300 species to 2006 (2).

All 6 fish species in this study are native and belong to Cyprinidae and Sisoridae families. The genus *Capoeta* has a wide distribution in Southwest Asia and contains about 20 species of which 7 occur in Iran (3). The barbells are also found in many water resources of Southwest Asia including Iran and comprise about 800 species with 15 formerly recognized in Iran. *Glyptothorax silviae* is reported only from rivers draining to the Persian Gulf in southwestern Iran in upper Karun and middle to lower Khersan and Armand Rivers in the Tigris basin (3, 4).

Armand River, also known as Karun Olia, is one of the main branches of Karun River, with 200 km length and 9983 square meter basin which finally along with the Karun River join Persian Gulf. The aforementioned river has been paid little attention disregarding its ecologic importance. The only study about this river is the one in 1999 by Ghorbani which had lead to the finding of 10 species of fishes belonging majorly to Cyprinidae family (3). This study was therefore undertaken to determine the prevalence of parasites in fish from Armand River, Chaharmahal va Bakhtyari Province, Iran.

## Materials and Methods

### Fish hosts

A total of 279 individual fish specimens from 6 species including *Capoeta aculeata* (n = 50), *C.damascina* (n = 126), *C. capoeta capoeta* (n = 14), *Barbus barbulus* (n = 69), *B. grypus* (n = 8), *and Glyptothorax silviae* (n = 12), were examined between autumn 2009 and summer 2010. Fishes were caught by local fisherman using gill nets or by angling and were carried alive to the laboratory in aerated tanks of water. In the laboratory, each specimen was individually measured for the total and standard length, weight, age and sex (Table 1). Identification of the fish species was made according to Coad, 1992 (3).

**Table 1:** Age, sex, weight and length of studied fishes

Fish species	No. of fish		Weight (g)		Age (yr)		Total length (cm)	

	Male	Female	Range	Mean±SD	Range	Mean±SD	Range	Mean±SD
*Capoeta aculeata*	25	25	112.4-723	319.9±153.3	1-4	2.9±0.72	22.3-43.5	32.1±5.48
*Capoeta damascina*	57	69	32.5-723	297.5±115.79	1-4	2.7±0.74	21-45	41.7±4.06
*Capoeta capoeta*	9	5	63.1-1285	301±415.5	1-4	2.2±0.80	19.2-55	29.6±11.37
*Barbus barbulus*	29	40	48.3-446	241.6±101.02	1-4	2.5±0.91	16-37.4	29.3±5.44
*Barbus grypus*	2	6	55.5-214.9	149.7±94.08	1-2	1.75±0.46	20-36	25.8±5.75
*Glyptothorax silviae*	3	9	28.4-356	210.5±102.7	1-3	2.25±0.66	15-31	27.1±6.15
Total	125	154	32.5-1285	279.9±151.3	1-4	2.6±0.81	15-55	30.7±5.56

### Sample preparation and Identification

A complete examination for parasites was done about each specimen. External surfaces of body, gills, eyes and internal organs as well as the entire body cavity and intestine were inspected for parasites. The collected parasites were preserved in 4% formaldehyde (Digenea, Cestoda and Crustacea), in a mixture of ammonium picrate and glycerin (Monogenea) or in a mixture of glycerin and alcohol (Nematoda). Parasites were identified in accordance with the keys given by Gussev, 1985; Lom and Dykova, 1992; Kabata, 1988; Jalali, 1997 and Moravec, 1998 (5–9), using a light microscope equipped with phase-contrast, differential interference contrast and Digital Image Analysis (Pro Plus 1.3).

### Statistical analysis

An analysis of variance (ANOVA) among the sampling seasons, biometric characteristics and host species was performed in order to test for the differences in parasite abundance between different fishes.

## Results

**Fig. 1:**
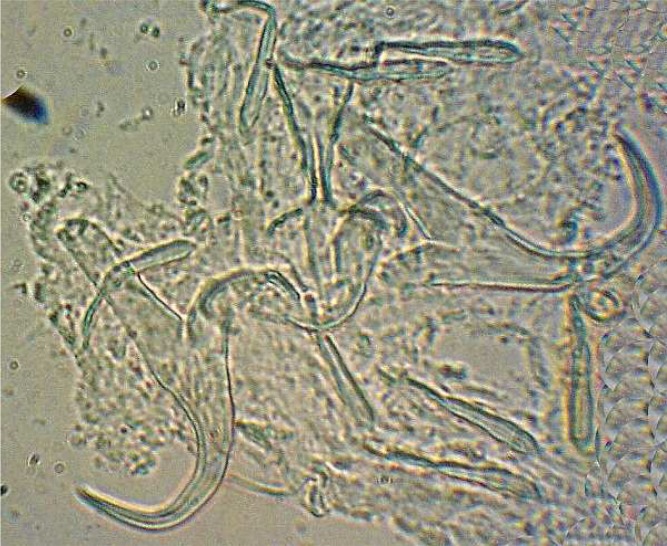
Opistohaptor of *Dactylogyrus akaricus*

**Fig. 2:**
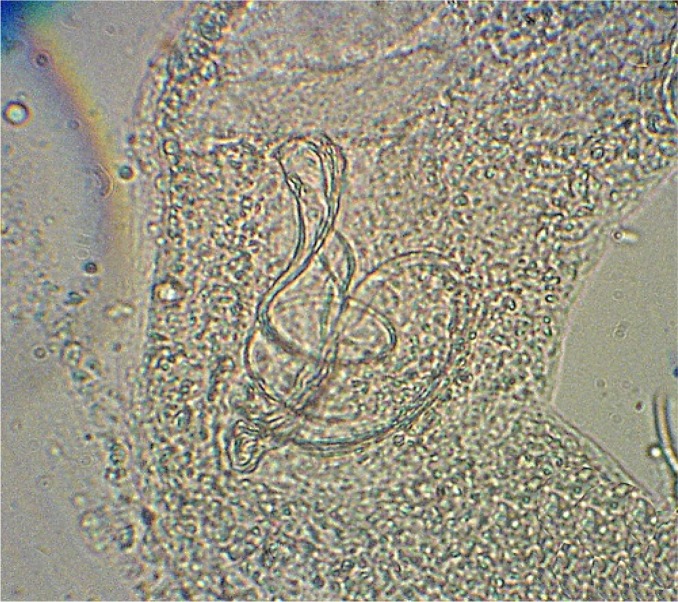
Reproduction organ of *Dactylogyrus akaricus*

**Fig. 3:**
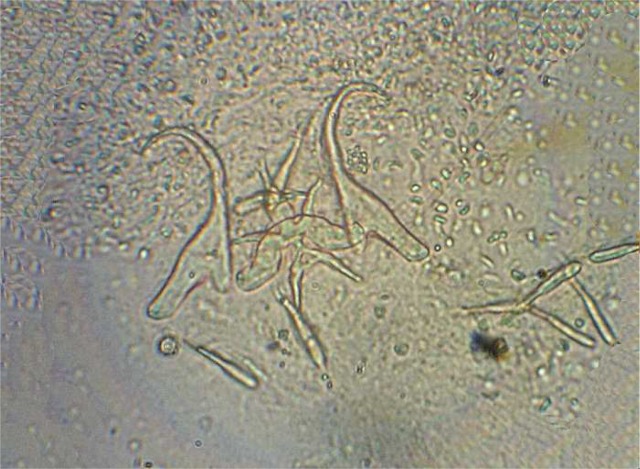
Opistohaptor of *Dactylogyrus skrjabiensis*

**Fig. 4:**
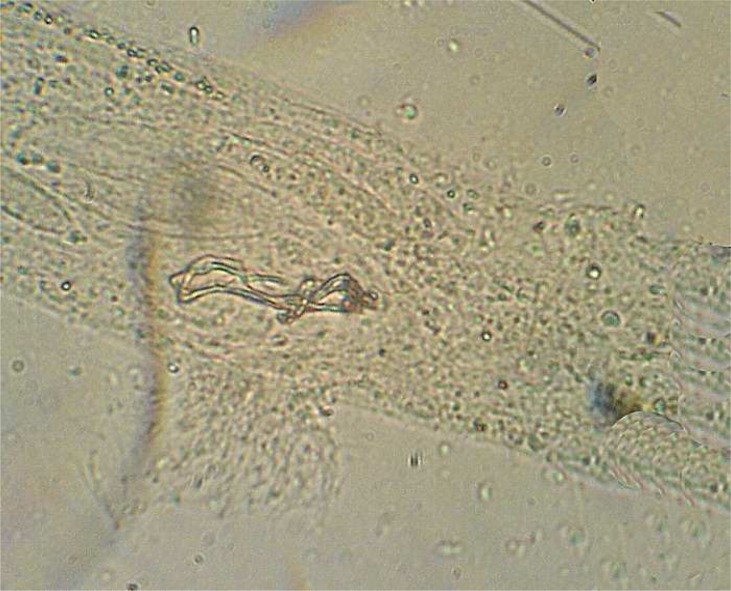
Reproduction organ of *Dactylogyrus skrjabiensis*

A total of 19 parasite species from 11 families in 6 fish species was recorded including: Ichthyophthirius multifiliis from cilliophora, Myxobolus musayevi from myxozoa, Dactylogyrus lenkorani, D. gracilis, D. pulcher, D. chramuli, D. akaraicus, D. skrjabiensis, Paradiplozoon sp. and two Gyrodactylus species from monogenea, Lamproglena compacta, Copepodid of Lernaea cyprinacea and Ergasilus sp. from crustacea, Allocreadium isoporum and A. pseudaspii from digenea, Kawia sp. and Bothriocephalus gowkongensis from cestoda and Rhabdochona denudata from nematoda. Biometric characteristics of studied fishes are shown in Table 1 and the parasites, their hosts and prevalence for each species are presented in Table 2. This inventory contributes 2 new host records (Dactylogyrus akaraicus and D. skrjabiensis in Barbus barbulus) and reports the presence of D. akaraicus and D. skrjabiensis in Iran for the first time. Opistohaptor and hooks of the new parasites are shown in Figures 1–4.

**Table 2:** Parasites of fishes in Armand River

Parasite group	Parasite species	Host	Infected Organ	Prevalence (%)
Ciliophora	*Ichthyophthrius multifilis* Fouquet, 1876	*Capoeta aculeata*	Gills and skin	62
		*Capoeta damascina*		52.3
		*Capoeta capoeta*		42.8
		*Barbus barbulus*		26
		*Barbus grypus*		25
		*Glyptothorax silviae*		16.6
Myxozoa	*Myxobolus musayevi* Kandilov, 1963	*Capoeta aculeata*	Gills	4
		*Capoeta damascina*		0.79
Monogenea	*Dactylogyrus lenkorani* Mikhailov, 1967	*Capoeta aculeata*	Gills	36
		*Capoeta damascina*		41.2
		*Capoeta capoeta*		35.7
		*Barbus barbulus*		5.7
		*Barbus grypus*		25
	*Dactylogyrus gracilis* Mikhailov, 1974	*Capoeta damascina*	Gills	3.17
	*Dactylogyrus pulcher* Bykowsky, 1957	*Capoeta damascina*	Gills	0.79
	*Dactylogyrus chramuli* Kojava, 1960	*Capoeta damascina*	Gills	1.58
	*Dactylogyrus akaraicus* Mikhailov, 1974	*Barbus barbulus*	Gills	7.2
	*Dactylogyrus skrjabiensis* Achmerov, 1954	*Barbus barbulus*	Gills	13
		*Capoeta capoeta*		7.14
	*Gyrodactylus* sp1 Diesing, 1850	*Capoeta aculeata*	Gills	8
		*Capoeta damascina*		3.7
		*Barbus barbulus*		1.4
	*Gyrodactylus* sp2 Diesing, 1850	*Capoeta aculeata*	Gills	4
		*Capoeta damascina*		1.58
	*Paradiplozoon* sp. Achmerov, 1974	*Capoeta damascina*	Gills	0.79
Digenea	*Allocreadium isoporum* Loss, 1894	*Capoeta aculeata*	Intestine	2
		*Capoeta damascina*		0.79
		*Barbus barbulus*		1.4
	*Allocreadium pseudaspii* Loss, 1894	*Capoeta damascina*	Intestine	0.79
		*Barbus barbulus*		1.4
Crustacea	Copepodid stage of *Lernaea cyprinacea* Linnaus,1758	*Barbus barbulus*	Gills	2.8
	*Lamproglena compacta* Markevich, 1936	*Capoeta aculeata*	Gills	8
		*Capoeta damascina*		4.76
		*Barbus barbulus*		7.2
	*Ergasilus* sp. Nordman, 1832	*Barbus barbulus*	Gills	4.3
Cestoda	*Kawia* sp. Hsu, 1935	*Barbus barbulus*	Intestine	2.8
	*Bothriocephalus gowkongensis* Yeh, 1955	*Barbus barbulus*	Intestine	2.8
Nematoda	*Rhabdochona denudata* Dujardin, 1845	*Capoeta aculeata*	Intestine	52
		*Capoeta damascina*		49.2
		*Capoeta capoeta*		28.5
		*Barbus barbulus*		40.5
		*Barbus grypus*		37.5
		*Glyptothorax silvieae*		66.6

A total of 178 fish (63.7%) out of 279 studied fish were infected with parasites. The highest infection rate was observed in *C. aculeata* with 80% (40/50) and the lowest was in *B. barbulus* with 47.8% (33/69). The infection rate in other species was 75% (6/8) in *B. grypus*, 71.4% (10/14) in *C. capoeta*, 63.4% (80/126) in *C. damascina* and 75% (9/12) in *G. silviae*. In terms of the number of taxa recovered from the examined fish, monogenea was the most abundant group with 8 species. The most parasite abundance was found in *Capoeta damascina* and *Barbus barbulus* both with 13 parasite species. *Rhabdochona denudata* and *Ichthyophthirius multifiliis* were the most widely distributed parasites among the examined hosts; these species were found infecting all the host species. The other frequent species was *Dactylogyrus lenkorani*, found infecting all fish species except *G. silviae*.

The infection was found in 46/69 fish (66.6%) in autumn 2009, 41/61 fish (67.2%) in winter 2009, 54/72 (75%) in spring 2010 and 33/77 (42.8%) in summer 2010, which with no significant difference (*P*<0.05).

The percent of infection in males and females was 65% and 62.5%, respectively and no statistical relation was found between infection with sex, age and biometric characteristics of fish host.

## Discussion

A total of 6 of the 10 native fish species recorded by Ghorbani, 1999 in the Armand River were examined. Those species not examined in this study are *Alburnus alburnus*, *Barbus kosswigi*, *Garra rufa* and *Chondrostoma regium* and fish species *Barbus grypus* and *Capoeta capoeta* are reported for the first time in the river. None of the 6 examined fish species had been previously studied for parasites in Armand River. Out of the 19 recorded species 14 parasite species were collected from external organs and 5 species were collected from intestine. Thirteen parasites (68.4%) are autogenic, which implies that they mature in fish and their entire life cycle are completed within aquatic ecosystems. The results showed that 178/279 studied fish (63.7%) are infected with parasites. The infection rate was significantly higher in *C.aculeata* while the most parasite diversity was found in *C. damascina*. The difference in infection rate in studied fish species may be due to differences in biology, nutrition, behavior of fish and also environmental conditions.

There are different views on the effect of length and weight of the fish on parasitic infection rate. In some studies, smaller fishes had more parasitic infection rate (10–12) while some other researchers believe infection rate increases with increasing weight and length (13, 14). No statistical relation was found between the infection rate and biometric characteristics of the examined fish in this study.

Among the groups of parasites were found in this study, monogeneans presented the highest number of species. Monogenea is the group that has presented the greatest number of species so far. Monogeneans are a diverse group of parasites that exhibit a relatively high degree of host specificity comparing to other groups of parasites (8). The selection of certain host species by monogenean must be involved mainly with factors in the host surface. Thus, chemical stimuli emitted from the host, mechanical and behavioral mechanisms have been suggested to explain this host specificity (8, 15). In this study 8 species of monogeneans were found in the fish with *Dactylogyrus lenkorani* as the most abundant one. This parasite is specific to genus *Capoeta* that had been previously collected from *C.aculeata* and *C.damascina* (2, 14, 15) and *Barbus lacerta* (5). In spite of the fact that monogenean parasites possess high host specifity, they could be found temporarily in a host different from specific host. This could be because of the high similarities between specific host and main host (8). This can be also true about *D. skrjabiensis* which is specific parasite to *Barbus* genus that is collected from *Capoeta capoeta* in this study.

*Ichthyophthrius multifiliis* and *Rhabdochona denudata* which have low specifity were found in all fish species with 40.5% and 47.6% of infection, respectively.

*Rhabdochona denudata* was previously collected from several fish species in Khuzestan Province (16). The disease due to *I. multifiliis*, commonly known as Ichthyophthiriasis or white spot disease, is widespread and has been reported from different freshwater fish species in Iran (2, 8, 17). Ichthyophthiriasis is recognized as one of the most pathogenic diseases of fish resulting in significant economic losses in the affected fish species (18). Severe damages of the gills and skin epithelium occur due to the break of the parasites through host skin and gill during infection. This damage might lead to concession of osmoregulatory process and ion regulation leading eventually to death of host fish (8). For example, natural outbreak of the Ichthyophthiriasis was blamed for the deaths of 18 million *Orestias agassi* in Lake Titicaca, Peru (19). High infection rate with this parasite will have negative effect on native fish population in Armand River.

To understand the role of the community of parasites in an ecosystem, previous knowledge of the species composing them is required. Continuing such studies using taxonomic and systematic approaches is the key to understanding of how biotic and abiotic factors affect fish species, since there is no way to understand the effects on native fish population without knowing the parasites species.

In this vein and considering fish's high infection with some kinds of parasite, frequent investigation of water resources to identify the threatening factors and to preserve the native fish's generation utilizing some particular methods like biological control seems to be necessary.
